# Screening for Modifiable Risk Factors of Noncommunicable Diseases in Urban Young Adults, Indore, 2023–2024

**DOI:** 10.7759/cureus.86179

**Published:** 2025-06-16

**Authors:** Rajesh Kothari, Vinita Kothari, Abhirup Datta, Shubhi Tiwari, Ameya Vaze

**Affiliations:** 1 Emergency Medicine, Amaltas Hospital, Dewas, IND; 2 Pathology, Central Lab, Indore, IND; 3 Astronomy, Astrophysics, and Space Engineering, Indian Institute of Technology, Indore, Indore, IND; 4 Epidemiology and Public Health, Central Lab, Indore, IND

**Keywords:** blood cholesterol, blood sugar, body mass index: bmi, descriptive epidemiology, hypertension, mass screening, modifiable risk factors, non-communicable disease (ncd), obesity, young adults

## Abstract

Introduction: Noncommunicable diseases (NCDs) lead to huge mortality in the population under 70 years of age at a global level. A national program called the National Programme for Prevention and Control of NCDs (NPNCD), targeting mainly individuals over 30 years of age, has been launched in India. Nearly 200 million Indians are young adults (ages 18-30 years). The levels of modifiable risk factors in this young adult population, as well as the prevalence of NCDs, remain undocumented. We conducted this study to identify the extent of modifiable risk factors among individuals aged 18-30 years in Indore.

Methods: We conducted a cross-sectional study in Indore between 2023 and 2024. We planned to screen 10,000 young adults, based on a pre-calculated sample size table, by organizing camps in educational institutes. The paper-based tool collected data on sociodemography, behavioral risk factors, family history, dietary recall, and anthropometry. The samples were tested in a National Accreditation Board for Testing and Calibration Laboratories (NABL)-accredited lab in Indore. The biochemical tests conducted were fasting blood glucose, total serum cholesterol, serum creatinine, and serum glutamate pyruvate transaminase (SGPT). We performed descriptive epidemiology and analyzed the data for correlation using the chi-square test.

Results: The median (range) age of screened individuals was 20 (18-30) years. The median (IQR) BMI was 20 (18-22.4). Approximately 46% (4607) of individuals had elevated systolic blood pressure. Among females, 44% (2483) had elevated systolic blood pressure, whereas it was 50% (2199) among males. Abnormal blood glucose levels were observed in 4.0% (398), abnormal cholesterol levels in 5.3% (528), abnormal creatinine levels in 4.9% (491), and abnormal SGPT levels in 8.1% (809). The p-value for the correlation between tobacco consumption and elevated blood pressure, serum creatinine, serum cholesterol, and SGPT was <.05.

Conclusion: Approximately 25% of the screened individuals were classified as underweight, while fewer than 25% were overweight. About 50% exhibit hypertension, with a higher prevalence in males. Individuals who have elevated blood pressure, serum creatinine, cholesterol, and SGPT levels exhibit an increased likelihood of tobacco consumption. We need to screen more young adults for modifiable risk factors and develop strategies to help them in mitigating these risks.

## Introduction

Noncommunicable diseases (NCDs) or chronic diseases affect individuals due to a web of causation. The main risk factors, in addition to age and genetics, are tobacco, mainly smoking, excessive use of alcohol, or metabolic risk factors like raised blood pressure (BP), overweight/obesity, high blood glucose levels, and abnormal blood lipids [1]. The most common NCDs are cardiovascular diseases (such as heart attacks and stroke), cancers, chronic respiratory diseases (such as chronic obstructive pulmonary disease and asthma), diabetes, non-alcoholic fatty liver disease (NAFLD), and chronic kidney disease.

NCDs are a global problem, and their prevalence and burden are increasing. This also places significant pressure on the healthcare system. When NCDs affect a population younger than 70, they cause premature mortality and also hurt the economy and productive ability of a society [2]. In 2021 alone, 43 million people succumbed to NCDs, and out of those, nearly 18 million were people below the age of 70 years [3].

Like all the other disease control measures, controlling and targeting NCDs should also start with surveillance. This surveillance should include both diseases and the presence of risk factors. For this reason, the government of India has launched a program called the National Programme for Prevention and Control of NCDs (NPNCD) [4]. With this program, the government, in addition to opportunistic screening at facilities, also launched population-based screening for individuals aged 30 years and above.

A significant portion of the Indian population falls within the 18- to 30-year-old age group. By 2025, experts predict that this young adult population will exceed 200 million [5]. This age group also happens to be the most productive population for any country. Many studies have indicated that the approach to targeting and controlling NCDs in youths should be multifaceted. However, most strategies stop at health promotion activities and do not push for early detection.

The young population suffers from genetic predisposition, family history, and air pollution, in addition to years of harmful lifestyle and at-risk behavior. Insufficient awareness and screening lead to a significant increase in severity. We need strategies that target this younger population to identify the extent of at-risk behavior and the prevalence of modifiable risk factors and NCDs among them.

Hypertension, obesity, smoking and tobacco consumption, consumption of abusive levels of alcohol, high salt, sugar, or fat intake in the diet, high cholesterol levels, high glucose levels as in diabetics or prediabetics, and lack of physical activity are considered modifiable risk factors [6,7]. If these behaviors persist over a longer duration, they can lead to various NCDs. Documentation of the prevalence of these risk factors among young Indian adults is imperative. Indore is the financial capital of Madhya Pradesh. It has also been the cleanest city in India for the last seven years. An increasing number of young adults are settling in Indore either for higher education or for jobs. With the changing lifestyle and increasing young adult population in Indore, information about NCDs and their risk factors is ever more crucial.

The study identified this gap and aimed to estimate the prevalence of modifiable risk factors for NCDs, including elevated BP, biochemical parameters, body mass index (BMI), and behavioral habits, among urban young adults aged 18-30 years in Indore.

## Materials and methods

Study design and setting

The study was conducted as a cross-sectional study in urban Indore in 2023. Camps were organized to recruit study participants from various colleges and institutes, including those specializing in engineering, art, science, management, and combined campuses. The sampling recruited each volunteer who gave consent for the study and was in the age group of 18-30 years. The volunteers were encouraged to invite their friends and colleagues to get screened as well.

Sample size

Using a pre-calculated table, with considerations for a 1% margin of error, a 95% confidence level, a 50% proportion of at least one modifiable risk factor, and a population estimate of 1,000,000 young adults in Indore, the sample size was calculated as 9513 [8]. This was then rounded off to 10,000 young adults in Indore. Young adults were defined as any individual in the age group of 18-30 years [9].

Inclusion and exclusion criteria

Anyone who provided consent was considered eligible. Anyone who could not give consent due to a mental disorder was excluded.

Data collection and tool

Data were collected in a paper-based format. The tool was a modified version of the WHO STEPS surveillance instrument and the community-based assessment checklist for NCD used by the government of India [10,11]. Data were collected on sociodemographic characteristics, behavioral risk factors, and anthropometry. Anthropometric measurements included height, weight, BMI, and waist circumference. We also examined their BP, which is a marker for many NCDs.

Anthropometric and BP measurement

Height, Weight, and Waist Circumference

In the camp setup, height was measured by sticking a scale on the wall and asking the participants to stand with their backs against a straight wall. The height was measured in meters (m). For the measurement of waist circumference, a standardized, stretch-resistant tape was used, following the guidelines provided by the WHO STEPS surveillance protocol [12]. The location was at the midpoint between the lower margin of the last palpable rib and the top of the iliac crest and was recorded when the tape was snug around the waist. The circumference was recorded in inches (in). The normal waist circumference for males was below 1.02 m, whereas it was below 0.88 m for females [13].

A calibrated digital weighing scale was used to measure weight. The participants were asked to stand on the scale without shoes and accessories. The weight was recorded in kilograms (kg).

Using height and weight, BMI is calculated. BMI is equal to weight (in kg) divided by the square of height (in meters). We defined the normal BMI range as between 18.5 and 24.9 kg/m, classifying 25-29.9 as overweight and >30 as obese [13].

To record BP, every participant was required to sit down in a chair. Subsequently, a trained nurse recorded the BP using a calibrated digital BP monitor. Standardized BP was considered as per the American Heart Association recommendations [14].

Blood sample collection method and biochemistry

From every individual, 2-3 mL of blood was collected with an appropriate anticoagulant. The samples were transported in triple-layer packaging with proper labeling. Sample request forms were attached and sent for testing at a National Accreditation Board for Testing and Calibration Laboratories (NABL)-accredited lab in Indore. The biochemical tests conducted were fasting blood glucose, total serum cholesterol, serum creatinine, serum protein, serum albumin, serum globulin, and serum glutamate pyruvate transaminase (SGPT). The standard values are given in the results table. The reference ranges are derived from the article published by Sairam et al. for the national reference [15].

Quality assurance

The samples were collected in accordance with Good Laboratory Practices, maintaining all aseptic conditions. The biomedical waste (BMW) generated was also disposed of with proper precautions and in accordance with BMW management guidelines [16,17]. Data were anonymized before analysis using laboratory IDs.

Data management and analysis

Data were entered into MS Excel (Microsoft Corporation, Redmond, Washington), and duplicates were removed by comparing names, age, gender, and phone number as parameters. The participants whose blood samples could not be processed were removed from analysis.

The study performed descriptive epidemiology, using simple ratios and proportions [18]. The data were also analyzed for correlation using the chi-square test. A p-value of <0.05 at a 95% confidence level was considered statistically significant. Odds ratios, along with 95% confidence intervals, were also calculated. QGIS was used to analyze the place distribution of sites where camps were organized. MS Excel and Epi Info (CDC, Atlanta, USA) were the software used for analysis.

Ethical considerations

The study was approved by the Institutional Ethics Committee, Amaltas Institute of Medical Sciences, Dewas (approval no. ECR/1145/INST/MP/2018/RR-22). All participants were provided with information before joining the study, and their informed consent was obtained. They also had the right to reconsider their participation at any time. Personal identifying information, such as name, contact details, and address, was hidden using unique IDs and was not provided to the data analysis team. The study adhered to the Declaration of Helsinki and the National Ethical Guidelines for Biomedical and Health Research 2017, as established by the Indian Council of Medical Research.

## Results

**Figure 1:**
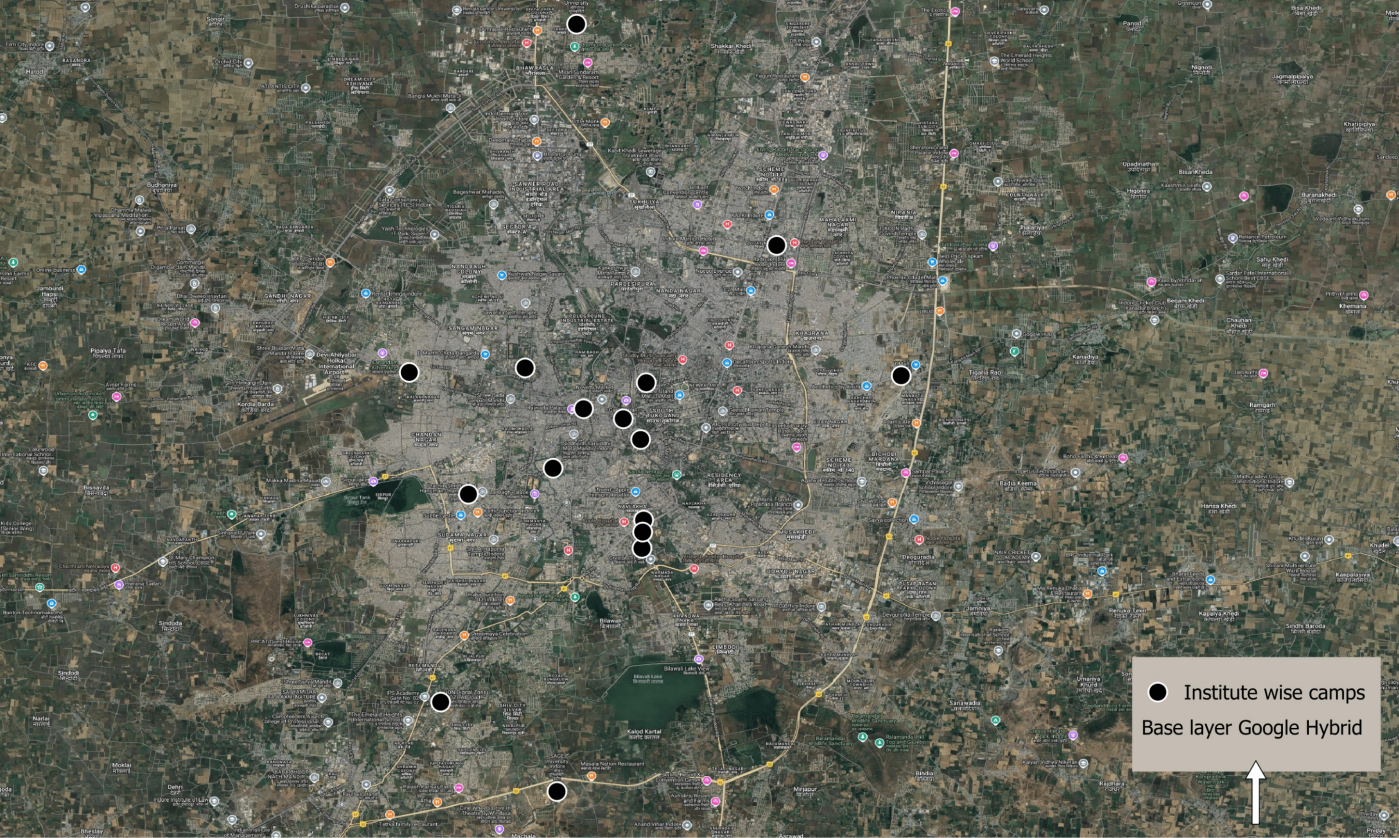
Institute-wise camp sites, Indore 2022–2023 Image Credits: Dr. Ameya Vaze, created using QGIS, with freely available vector Google satellite map and spot map generated from lat-longs collected from the campsite.

We conducted camps across 17 colleges and institutes and screened 10,002 individuals. Figure 1 illustrates the distribution of these colleges throughout the urban Indore area.

Demography of participants

**Figure 2:**
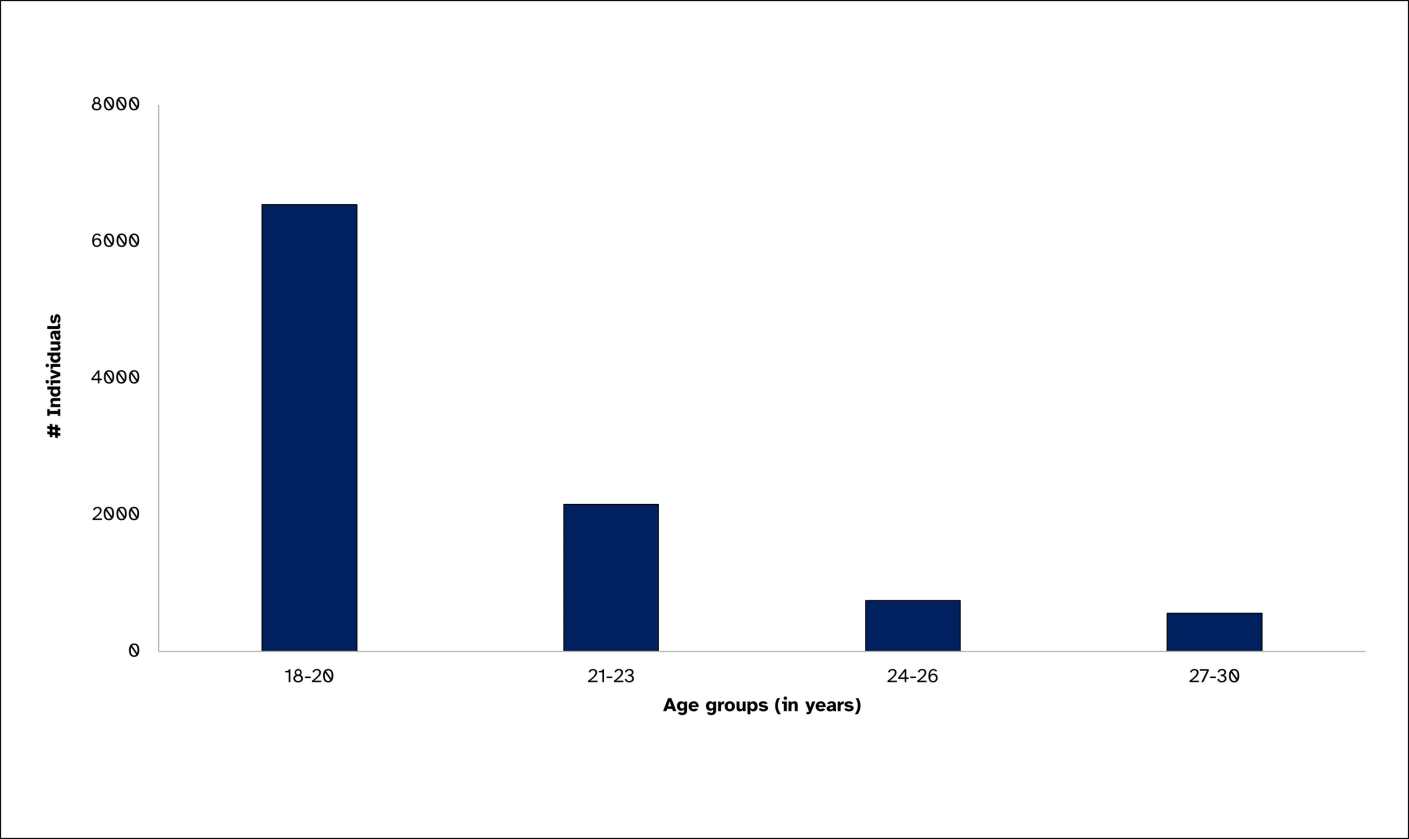
Age group-wise distribution, urban young adults, Indore, 2023 (N=10,002)

The median age of study participants was 20 years (18-30). As shown in Figure 2, most individuals, i.e., 65% (6542), were between the ages of 18 and 20 years. Out of all the participants, 56% (5628) were females. The correlation between male sex and BP, cholesterol, creatinine, and SGPT is statistically significant (Table 1).

**Table 1:** Correlation (p-value) between BMI, tobacco consumption, and male sex and modifiable risk factors, young adults, Indore, 2023–2024 SGPT: Serum Glutamic Pyruvic Transaminase; BMI: Body Mass Index

Independent variable	Elevated systolic blood pressure	Elevated blood glucose	Elevated creatinine	Elevated cholesterol	Elevated SGPT	Lower protein
BMI (p-value)	0.2	0.16	0.00	0.00	0.00	0.18
Tobacco	0.04	0.13	0.00	0.00	0.00	0.70
Sex (male)	0.00	0.91	0.00	0.00	0.00	0.15

Anthropometry and BP

**Figure 3:**
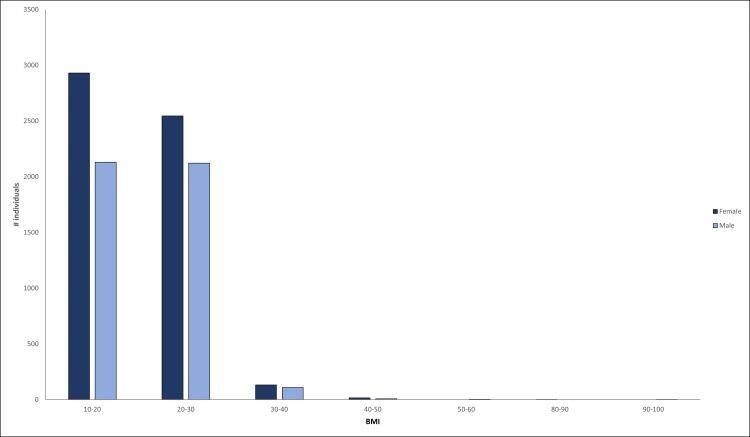
BMI vs. gender, young adults, Indore, 2023

The median height of male participants was 168 cm (72-213 cm), whereas the median height of female participants was 157 cm (79-198 cm). The median weight for male participants was 55 kg (27.5-129 kg), and the same for female participants was 48 kg (25-102 kg). The median (range) waist circumference was 30 inches (20-47). Waist circumference above 35 inches was observed in 0.5% (27) of females, and above 40 inches was observed in 0.2% (8) of males. The median (IQR) BMI was 20 (18-22.4), and the gender-wise proportions are shown in Figure 3, with a BMI of <20 observed among 52% (2931) of females and 49% (2130) of males, respectively. Overweight individuals were 9.6% (962), obese individuals were 2.7% (274), whereas underweight individuals were 30% (3071). Table 1 presents the correlation between BMI and BP, as well as other biochemical tests. The correlation between BMI and cholesterol, creatinine, and SGPT is statistically significant.

The median (range) systolic BP was 115 mmHg (87-198 mmHg). Approximately 44% (4434) of individuals had elevated systolic BP (between 120 and 129 mmHg), 2% (172) had hypertension stage 1 (between 130 and 139 mmHg), whereas 1% (76) had hypertension stage 2 (140 mmHg or higher). The median (range) diastolic BP was 68 mmHg (50-122 mmHg). Around 12% (1160) of individuals had stage 1 hypertension based on diastolic BP (80-89 mmHg) measurement. Additionally, 0.1% (14) had hypertension stage 2 as per diastolic BP (90 mmHg or higher). Nearly 35% (3529) of individuals had either a wide or narrow pulse pressure (less than 40 or more than 60 mmHg). Among females, 44% (2483) had systolic BP above 120 mm of Hg, while among males, it was 50% (2199). Table 1 displays the correlation results between sex (male) and elevated systolic BP. This correlation is statistically significant.

Biochemistry

Abnormal biochemistry level proportions are shown in Table 2. The median (range) glucose was 85.1 mg/dL (52.1-529). Among females, 4% (225) and among males, 4% (173) had blood glucose levels elevated above 110 mg/dL. The median (range) cholesterol levels were calculated as 145.1 mg/dL (60-341.9). Among females, 4.1% (233) and among males, 6.74% (295) had blood cholesterol levels elevated above 200 mg/dL. The median (range) creatinine levels were calculated as 0.83 mg/dL (0.26-2.82). Among females, 1.1% (64) and among males, 9.76% (427) had blood creatinine levels elevated above 1.2 mg/dL. The median (range) SGPT was calculated at 20 U/L (4.7-1457). Among females, 3.8% (218) and among males, 13.5% (591) had blood SGPT levels elevated above 41 mg/dL. The median (range) protein was calculated at 20 g/dL (4.7-1457). Among females, 0.37% (21) and among males, 0.39% (17) had blood protein levels below 6.4 mg/dL. Table 1 presents the correlation results between male sex and all the biochemical abnormalities.

**Table 2:** Abnormal biochemical test results, young adults, Indore, 2023 SGPT: Serum Glutamic Pyruvic Transaminase

Variable	Participants with abnormal test results, n	Proportion (%)
Glucose (>110 mg/dL)	398	4.0%
Cholesterol (>200 mg/dL)	528	5.3%
Creatinine (>1.2 mg/dL)	491	4.9%
SGPT (>41 U/L)	809	8.1%
Protein (<6.4 g/dL)	38	0.38%

Behavioral risk

The proportion of reported consumption of tobacco was only 1.5% (159). Among females, 0.11% (6) and among males, 3.5% (153) reported consuming tobacco. Elevated blood glucose levels were observed in 6.2% (10) of individuals who consumed tobacco and in 3.9% (388) of those who did not consume tobacco. Elevated systolic BP was observed in 54.7% (87) of tobacco smokers and in 46.7% (4595) of those who reported that they do not consume tobacco. The correlation results between tobacco consumption and blood glucose and between tobacco consumption and systolic BP, as p-values, are shown in Table 1. The correlation between tobacco consumption and BP, cholesterol, creatinine, and SGPT is statistically significant.

Only 4% (405) disclosed their alcohol consumption. Among females, 0.34% (19) and among males, 8.8% (386) reported consuming alcohol. The total number of individuals reporting consumption of a non-vegetarian diet was 12.5% (1250). Table 3 presents the correlation results between BMI and fasting glucose, as well as between BMI and blood pressure.

**Table 3:** Odds ratio between BMI, tobacco consumption, and male sex and modifiable risk factors, young adults, Indore, 2023 SGPT: Serum Glutamic Pyruvic Transaminase; BMI: Body Mass Index; CI: Confidence Interval

Independent variable	Elevated systolic blood pressure odds ratio (95% CI)	Elevated blood glucose odds ratio (95% CI)	Elevated creatinine odds ratio (95% CI)	Elevated cholesterol odds ratio (95% CI)	Elevated SGPT odds ratio (95% CI)	Lower protein odds ratio (95% CI)
BMI (p-value)	11.1 (0.9-1.2)	1.2 (0.9-1.6)	1.5 (1.1-1.8)	2.1 (1.7.2.6)	2.1 (1.7-2.5)	0.4 (0.1-1.6)
Tobacco	1.4 (1-1.9)	1.6 (0.8-3.1)	3.6 (2.3-5.6)	2.5 (1.5-4.1)	2.9 (1.98-4.36)	3.5 (0.8-14.5)
Sex (male)	1.28 (1.2-1.4)	0.99 (0.8-1.2)	9.4 (7.2-12.3)	1.7 (1.4-2.0)	3.9 (3.3-4.6)	1.0 (0.5-2.0)

## Discussion

A study conducted by Bhandari et al., based on the 2016 National Family and Health Survey (NFHS) data, showed that approximately one-fifth of the population aged 15 years and above is underweight [19]. This study shows a similar and alarming trend that nearly one-third of the young adults in Indore are underweight. However, similar comparisons to the overweight populations were not observed. Bhandari et al. reported that almost a fifth of the population is overweight, whereas in the present study, less than a tenth of the study participants were overweight.

Ramakrishnan et al., in their comprehensive Indian BP survey, documented that among Indians 18 years and above, slightly less than one-third of the population is hypertensive [20], which is not reflected in this study, which only covers the population in the 18-30 age group. Even in the age group of 18-44 years, the prevalence of hypertension was slightly less than a fifth. In this present study, fewer than three out of 100 screened individuals had either stage 1 or stage 2 hypertension. Ramakrishnan et al.'s study also showed that nearly half of the participants were pre-hypertensive, with elevated BP, and this trend is reflected similarly in this study. According to the results of this study, slightly less than half of the young adults suffer from elevated BP. Ramakrishnan et al. also discussed the prevalence of elevated BP being higher than in other population groups, such as in the United States. A similar issue was noted by Roy et al., who found that slightly less than a third had undiagnosed hypertension, but it was conducted in the age group of >30 years [21]. Similarly, Geevar et al. also showed that nearly a third of those screened had elevated BP [22].

Lal et al., in their study based on analysis of NFHS 5 data, also showed that nearly 40 individuals out of 100 in the 15-30 age group have either narrow or wide pulse pressure [23]. This study also shows that almost 35 out of 100 individuals have a similar wide or narrow pulse pressure.

As compared to the other studies, the consumption of tobacco, based on self-report, was much smaller in this study. Only four individuals per 100 screened reported consuming tobacco, whereas this proportion ranges between 18% and 35% in other studies [9,21,22,24]. According to a study published by Yadav et al., based on NFHS data, the population in the 20-29 age group has lower odds of consuming tobacco compared to older adults. It also indicated that the overall trend of tobacco consumption has decreased, with only 8% of people consuming tobacco, according to NFHS 5 [25]. The study also noted that underreporting, which may be due to stigma and other factors, could explain the low reporting rate. In the current study, the camps were conducted on college premises, with faculty members participating in the organizing committee. This might be a reason for the underreporting of tobacco and alcohol consumption in this study.

Sia et al., in their retrospective cohort study, found that smoking was associated with glycemic control, and this study did not find a statistically significant association between smoking and elevated blood glucose level [26].

Limitation

This study was cross-sectional in design. The sampling strategy was purposive sampling, which means samples were not necessarily equally distributed from all areas of Indore. The sampling was conducted in educational institutions; hence, a large sample consisted of students from the 18-20 age group. Due to self-reporting in front of the faculty organizing the camps, there might have been underreporting of consumption of tobacco and alcohol.

## Conclusions

This study showcases that over two-thirds of those screened were 20 years or younger, nearly a third were underweight, nearly half had elevated systolic BP, and less than a tenth of those screened had biochemical abnormalities. The study also concluded that individuals who have elevated serum creatinine, cholesterol, and SGPT have higher odds of having a higher BMI. Individuals who have elevated BP, serum creatinine, cholesterol, and SGPT have higher odds of consuming tobacco. Individuals who have elevated BP, serum creatinine, cholesterol, and SGPT have higher odds of being male.

Considering the burden of elevated BP in young urban adults, studies are needed to establish causality and identify feasible interventions that can help reduce this burden, not only in the population but also at the individual level. In addition to surveys on modifiable risk factors of NCDs for the urban young adult population, surveys to recognize the extent of undernutrition and malnutrition should also be considered. This will provide an in-depth understanding of the diet being consumed and the level of intervention required to modify it. Additionally, the causes behind the prevalence of modifiable risk factors require further study. Good evidence will lead to a better policy to tackle these problems. Since Indore is the economic capital of Madhya Pradesh and an academic center in central India, similar studies can be conducted at other major academic centers to screen young adults for modifiable risk factors of NCDs. With this knowledge, programs targeting young adults to help them reduce BP levels and maintain a healthy BMI and other biochemical risk factors should be launched in colleges and educational institutions.
